# Membrane feeding of dengue patient’s blood as a substitute for direct skin feeding in studying *Aedes*-dengue virus interaction

**DOI:** 10.1186/s13071-016-1469-6

**Published:** 2016-04-15

**Authors:** Cheong-Huat Tan, Pei-Sze Jeslyn Wong, Mei-Zhi Irene Li, Hui-Ting Yang, Chee-Seng Chong, Linda K. Lee, Shi Yuan, Yee-Sin Leo, Lee-Ching Ng, David C. Lye

**Affiliations:** Environmental Health Institute, National Environment Agency, Singapore, Singapore; Faculty of Science, Monash University, Melbourne, Australia; Communicable Disease Center, Tan Tock Seng Hospital, Singapore, Singapore; School of Biological Sciences (SBS), Nanyang Technological University, Singapore, Singapore

**Keywords:** Dengue, *Aedes aegypti*, Direct Skin Feeding Assay (DFSA), Membrane Feeding Assay (MFA), EDTA blood

## Abstract

**Background:**

Understanding the interaction between *Aedes* vectors and dengue viruses (DENV) has significant implications in determining the transmission dynamics of dengue. The absence of an animal model and ethical concerns regarding direct feeding of mosquitoes on patients has resulted in most infection studies using blood meals spiked with laboratory-cultured DENV. Data obtained from such studies may not reflect the natural human-mosquito transmission scenario. This study explored the potential of using membrane feeding of dengue patient’s blood as a substitute for direct skin feeding.

**Methods:**

Four to six-day old female *Ae. aegypti* were provided the opportunity to feed via direct exposure to a patient’s forearm for 15 min or via exposure to EDTA-treated blood from the same patient through an artificial membrane for 30 min. Mosquitoes from both feeding methods were incubated inside environmental chambers. Mosquitoes were sampled at day 13 post-feeding. Midgut and salivary glands of each mosquito were dissected to determine DENV infection by RT-qPCR and viral titration, respectively.

**Results:**

*Feeding rates*: Direct skin feeding assay (DSFA) consistently showed higher mosquito feeding rates (93.3–100 %) when compared with the membrane feeding assay (MFA) (48–98.2 %). *Midgut infection*: Pair-wise comparison between methods showed no significant difference in midgut infection rates between mosquitoes exposed via each method and a strong correlation was observed in midgut infection rates for both feeding methods (*r* = 0.89, *P* < 0.0001). Overall midgut viral titers (*n* = 20) obtained by both methods were comparable (*P* ≥ 0.06). *Salivary gland infection*: Pair-wise comparison between both methods revealed no significant difference in salivary gland infection rate. Strong correlation in salivary gland infection was observed between DSFA and MFA (*r* = 0.81, *P* < 0.0001). In general, mosquitoes fed directly on dengue patients and those on patients’ blood (*n* = 11) had comparable virus titer (*P* ≥ 0.09).

**Conclusion:**

DENV midgut and salivary gland infection rates showed good concordance between DSFA and MFA blood meal exposure methods. Freshly-obtained venous blood in EDTA from dengue patients for MFA can be used as a substitute to DSFA, especially in circumstances where bioethics approval or patient recruitment is difficult to obtain for vector competence studies. Nevertheless, mosquito numbers will need to be increased to compensate for lower feeding rate in MFA.

**Electronic supplementary material:**

The online version of this article (doi:10.1186/s13071-016-1469-6) contains supplementary material, which is available to authorized users.

## Background

The natural cycle of dengue infection in *Aedes* spp. vectors begins when the mosquito feeds on a viremic host. Initial viral replication occurs in the midgut of the mosquito, followed by dissemination and replication of the dengue virus in various body parts, such as fat bodies, neural ganglia and the salivary glands [1]. Once dengue viruses (DENV) have reached the salivary glands, transmission of virus to susceptible hosts may occur during subsequent blood meals. This process is important in shaping the epidemiology of dengue and is greatly affected by a myriad of viral, mosquito, host and environmental factors. Studies of mosquito-virus interactions have generated important data that enhance our understanding of the epidemiology of dengue [2].

The role of *Aedes aegypti* in the transmission of dengue fever was first recognized by Bancroft [3] in 1906 by feeding mosquitoes on dengue patients. This initial finding was further corroborated by Cleland and colleagues using similar methods [4, 5]. More comprehensive human to mosquito transmission studies were conducted in the Philippines by Siler et al. [6] and Simmons et al. [7]. These classical human transmission studies prompted several epidemiologically important observations including (i) lifetime infection of dengue virus in mosquito vectors, (ii) infectivity of dengue patients in the late prodromal stage to *Ae. aegypti*, and (iii) confirmation that *Ae. albopictus* is also an important vector for dengue. To date, the largest and most comprehensive studies involving human volunteers were conducted by Nguyen et al. [8] in Vietnam which revealed that the majority of symptomatic, ambulatory dengue patients were important sources of infection for vectors and that DENV plasma viremia levels served as an important marker of the duration of human infectiousness to *Ae. aegypti.* Most importantly, the study defined serotype-specific viremia thresholds which must be reached by vaccines or drugs to prevent DENV transmission.

The value of studies based on direct feeding cannot be overemphasized as they reflect epidemiologic reality. However, the challenges in conducting such studies limit the approach. First, ethical approval may be difficult to obtain in some countries, and secondly recruitment of patients may be a challenge. Due to these limitations and the lack of a systematically viremic animal model for DENV, studies of dengue infection in vectors have largely been restricted to membrane feeding assay (MFA) using artificial infectious blood meals [2]. These usually involve feeding mosquitoes with vertebrate blood spiked with cell cultured virus, using a device, such as a “double-jacketed” glass cylinder [9] or a Hemotek membrane feeding system (Discovery Workshops, United Kingdom) [10]. However, these methods may not accurately mimic that of the natural setting as a much higher amount of virus is required to infect mosquitoes when using artificial methods compared with feeding directly on viremic hosts [11]. Earlier studies have also shown that infection rates were lower in mosquitoes that were infected with other viruses via artificial methods compared to those feeding directly on viremic hosts [12, 13]. These lower infection rates may be explained by the use of virus stock that had been frozen and thawed, or phenotypic virus adaptation due to numerous passages in cell culture [14–16].

In this study, we evaluated the use of membrane feeding of dengue patient’s blood as a substitute for direct skin feeding in *Aedes*-dengue virus interaction studies. The advantages and disadvantages of membrane feeding assay and direct skin feeding assay (DFSA) are discussed.

## Methods

### Patient recruitment and characterisation of viremia

#### Patients

Written informed consent was obtained from each patient. The inclusion criteria were: (i) adult, ≥ 21 years of age, (ii) ≤5 days of fever, and (iii) positive for DENV by point-of-care dengue NS1 Ag rapid test kit (Standard Diagnostic Inc, Korea). All patients were recruited from Tan Tock Seng Hospital, Singapore. Upon enrolment, 12 ml of blood were drawn from dengue patients by a trained phlebotomist into a sterile EDTA Vacutainer® tube (Beckton Dickinson, USA). The mosquitoes were then exposed to the EDTA blood within 10 min after the blood was withdrawn. All experimental procedures were conducted at the Environmental Health Institute (EHI), Singapore. The study was approved by the Domain Specific Review Board, National Healthcare Group, Singapore (NHG DSRB Ref: 2013/00111). The study was conducted from July to December 2013.

#### DENV serum viremia levels

DENV RNA was isolated from patient serum using the QIAamp Viral Mini Kit (Qiagen, Germany) following the manufacturer’s recommendations. The number of RNA copies in patient’s serum was measured using a TaqMan® one-step qRT-PCR assay targeting a highly conserved 3′UTR region of DENVs. Oligonucleotide sequences used were DENVF2: 5′- AAACAGCATATTGACGCTGGGA-3′ and DENVR3: 5′-GGCGYTCTGTGCCTGGAWTGATG-3′, with a probe sequence of 5′-FAM- AGACCAGAGATCCTGCTGTCTC-MGB-3′. PCR reactions were carried out using the TaqMan® Fast Virus 1-step Master Mix (Life Technologies, USA). The one-step qRT-PCR reactions were performed in a 20 μl reaction volume containing 5 μl of the extracted RNA, 1× master mix, and 0.5 μM each of the forward and reverse primers and 0.25 μM of the probe. Amplification was performed using the Rotor-Gene Q (Qiagen, Germany) according to the following programmes: one cycle each of 50 °C for 6 min and 95 °C for 20 s, followed by 45 cycles each of 95 °C for 3 s and 60 °C for 30 s. Amplification of the target gene from each individual patient was compared against a standard curve generated from 10-fold serial dilutions of an *in-vitro* transcribed dengue RNA standard.

#### Dengue serotypes

Dengue serotype from each patient was determined using a semi-nested PCR assay according to Lanciotti et al. [17] with modifications. Briefly, the first round of amplification reaction (dengue virus consensus primers D1 and D2), was performed using the one-step Access Quick™ RT-PCR System (Promega, USA) following the manufacturer’s recommendation with annealing temperature of 55^○^C. The product of this first reaction was then used as the template for the second amplification reaction using the upstream consensus primer D1 and serotype specific primers TS1, TS2, TS3 and TS4. The second nest reaction was performed using the GoTaq® Flexi DNA Polymerase (Promega, USA) following the manufacturer’s recommendation with the annealing temperature set at 55^○^C. The nest 2 amplicons were analysed by agarose gel electrophoresis, stained with GelRed (Biotium, USA) and observed using UV transilluminator.

### Human to mosquito transmission experiments

#### Mosquitoes

*Aedes aegypti* used for the experimental infections were derived from larvae collected from residential premises during routine inspections by enforcement officers of the National Environment Agency (NEA), Singapore. Only larvae collected from geographical locations with no dengue or chikungunya transmission were used. Larvae were reared in 25 cm × 30 cm × 9 cm enamel pans containing 800 mL of water and fed with Plecomin® fish food (Tetra, Germany). Pupae were placed in 30 cm × 30 cm × 30 cm (H × W × L) cages before emergence into adults Adults were allowed to emerge and were maintained under standard insectary conditions at 27 ± 1 °C and 75–80 % relative humidity (RH), with a photoperiod of 12 h:12 h light:dark (L:D) cycles. F_0_ mosquitoes were allowed to mate randomly and fed with pathogen-free pig’s blood (A*STAR Biological Resource Center, Singapore) using a Hemotek membrane feeding system (Discovery Workshops, United Kingdom) with mouse skin as membrane. The temperature of the feeding device was set to 37 ^○^C. F_1_ eggs were hatched in aged water. Larvae were reared and pupae were allowed to emerge as mentioned above. This process was repeated until an F_3_ generation was obtained. The F_3_ adults were used in the feeding experiments.

To ensure mosquitoes used in the study were not harbouring specific human mosquito-borne pathogens, at least 300 F_2_ parental lines were pooled into groups of 10–15 mosquitoes and screened for dengue virus, chikungunya virus [18], Zika virus [19], Ross River virus [20] and Pan-Flavi RT-PCR assays [21].

#### Direct skin feeding method

Seventeen to 34 mosquitoes (4–6 days old) were transferred into paper cups covered with net and were starved for at least 24 h before exposure to dengue patients. After the EDTA blood was collected, the cup was placed on patient’s forearm and the mosquitoes were exposed to feed through the net for 15 min. As an additional precaution against escapees, all direct skin feeding experiments were performed inside a glove-box, measuring 120 cm × 120 cm × 240 cm. The glove box was made of 0.5 cm thick acrylic plastic with three 60 cm diameter portholes where the patient and researcher’s arm were inserted (Additional file 1: Figure S1). The opening of the portholes was covered by elastic stockinet.

After exposure, the cups containing mosquitoes were immediately placed in double-layer containers and transported to the EHI arthropod containment level 2 (ACL-2) facility. Mosquitoes were cold anesthetized by placing the cups in ice and fully engorged females were transferred to new paper cups covered with net. Engorged females were maintained in an environmental chamber (Sanyo, Japan) set at a cyclical temperature between 29 and 31 °C and 70–80 % RH with a 12 h:12 h L:D cycle and provided with 10 % sugar/vitamin B complex *ad libitum*. The conditions provided in the environmental chamber simulate that of indoor conditions in Singapore (determined by placing data loggers inside naturally ventilated living rooms of eight homes randomly distributed across the island; data not shown).

#### Membrane-feeding method

Fifty to 60 mosquitoes (4–6 days old) from the same colony as above were transferred to 0.5 L cylindrical cardboard containers and were starved for at least 24 h before being exposed to dengue patient blood. EDTA tubes containing blood from dengue patients were placed inside a plastic container with armoured beads (Life Technologies, USA), pre-warmed to 37 °C, and immediately brought into the EHI ACL-2 facility where the blood was transferred into a Hemotek blood reservoir unit. Mosquitoes were then fed at a constant temperature of 37 °C using the Hemotek blood feeding system with mouse skin as membrane inside a feeding chamber acting as a secondary containment. After thirty minutes of exposure to patient’s blood, mosquitoes were cold anesthetized. Fully engorged females were transferred to paper cups covered with net and maintained as described above. This was carried out in tandem with DFSA.

### Processing of mosquitoes

#### Mosquito sampling

Up to 12 fully-engorged mosquitoes from each of the cohorts (MFA/DSFA) were sampled at day 13 post- feeding. Mosquitoes were knocked down on ice and midguts and salivary glands of each mosquito were dissected using the Medium 199 (Life Technologies, USA) and homogenized using a MM200 mixer mill (Retsch, Germany).

#### Detection of DENV in mosquito midguts using a qRT-PCR assay

Total RNA was amplified from mosquito midguts using the QIAamp Viral Mini Kit (Qiagen, Germany) following manufacturer’s recommendations. The number of RNA copies in mosquito midguts was estimated using the same TaqMan® one-step qRT-PCR assay.

#### Titration of DENV in mosquito salivary glands

Viral titers in mosquito salivary glands were determined using a Vero cell-based Tissue Culture Infectious Dose50 (TCID_50_) assay as described by Higgs et al. [22]. Briefly, samples were titrated in 10-fold serial dilutions in a 96-well microtititer plate and incubated with Vero cells at 37 °C and 5 % CO_2_. After a 7-day incubation, cells were fixed and stained with a monoclonal mouse anti-DENV (1 + 2 + 3 + 4) specific antibody (Immunology Consultants Laboaratory Inc, USA) and Vectastain ABC kit (Vector Laboratories, USA) following manufacturer’s protocol. All virus titers were expressed as Log_10_TCID_50_/ml.

### Statistical analysis

Feeding rates were calculated by dividing the number of mosquitoes that were fully engorged by the total number of mosquitoes exposed. The differences in the feeding rates between DSFA and MFA were analysed using paired Fisher’s exact tests. The midgut infection and salivary gland dissemination rates were calculated by dividing the number of infected midguts or salivary glands by the total number of organs analysed. Differences in midgut and salivary gland infection rates were also compared using paired Fisher’s exact tests. Kolmogorov-Smirnov tests indicated that the data did not conform to conditions of normality, hence non-parametric analyses were performed. The relationship of midgut and salivary glands infection rates between direct skin feeding and membrane feeding methods were analysed using the Spearman correlation coefficient tests. Differences in midgut viral genome copies and salivary gland viral titre between the two feeding methods were analysed using Mann–Whitney *U*-tests. All statistical tests were performed using MedCalc for Windows (MedCalc software, Belgium), with probability values of < 0.05 considered significant.

## Results

### Patients

A total of 26 dengue patients were recruited for the study. However, one patient requested that skin feeding be stopped halfway, due to discomfort, resulting in only partial or no blood meal in a majority of mosquitoes and exclusion from the study. Of the remaining patients, 20 (80 %) were found to be infected with DENV-1, three (12 %) were infected with DENV-3, and two (8 %) with DENV-2 (Table 1). The viremia level (DENV Log10 RNA copies/ml) of patients ranged from 4.75 to 9.41 (DENV-1), 3.73 to 5.6 (DENV-2) and 3.69 to 8.69 (DENV-3).

**Table 1:** Patient dengue virus (DENV) viraemia, serotype and midgut/salivary gland infection rates and virus titre in *Aedes aegypti* exposed using either direct skin feeding assay (DSFA) on patients or membrane feeding assay using EDTA-treated patient blood (MFA)

Patient	Serotype	Serum virus titre (log_10_ RNAcopy/ml)	Mode of infection	Feeding rate fed/*n* (%)	*P*-value^a^	Midgut			Salivary Glands		
						Infected/*n* (% infection)^b^	Number of RNA copies	*P*-value^a^	infected/*n* (% infection)^b^	DENV SG Titer (Log_10_TCID_50_/ml)	*P*-value^a^
5	1	9.49	DSFA	30/30 (100)	<0.0001*	100 (12)	9.07 ± 0.26	0.95	12/12 (100)	4.98 ± 1.03	0.16
			MFA	37/56 (66)		100 (12)	9.11 ± 0.13		12/12 (100)	5.52 ± 0.66	
8	1	9.21	DSFA	30/30 (100)	<0.0001*	9/9 (100)	9.16 ± 0.09	0.02*	9/9(100)	4.67 ± 0.94	0.02*
			MFA	27/56 (48.2)		12/12 (100)	8.45 ± 0.54		12/12(100)	5.24 ± 0.28	
9	1	5.65	DSFA	27/30 (90)	0.03*	12/12 (100)	3.62 ± 1.93	0.88	0/12	0	ND
			MFA	38/56 (67.8)		8/12 (66.7)	4.57 ± 3.10		0/12	0	
10	1	4.76	DSFA	30/30 (100)	0.007*	2/12 (16.7)	3.52	ND	0/12	0	ND
			MFA	45/56 (80.4)		2/12 (16.7)	3.08		0/12	0	
11	1	7.51	DSFA	29/30 (96.7)	0.25	12/12 (100)	6.22 ± 2.96	0.52	6/12 (50)	3.95 ± 0.7	0.09
			MFA	49/56 (87.5)		12/12 (100)	6.38 ± 3.3		5/12 (42)	4.95 ± 0.76	
12	1	9.18	DSFA	28/30 (93.3)	1	12/12 (100)	9.8 ± 0.48	0.52	12/12 (100)	5.18 ± 0.49	0.003
			MFA	51/56 (91.1)		12/12 (100)	9.66 ± 0.31		8/12 (67)	4.74 ± 1.66	
13	1	8.44	DSFA	17/17 (100)	0.57	12/12 (100)	9.69 ± 0.37	0.06	11/12 (92)	3.52 ± 1.19	0.82
			MFA	43/47 (91.5)		12/12 (100)	9.73 ± 1.51		12/12 (100)	4.89 ± -.66	
14	1	5.71	DSFA	17/17 (100)	0.001*	7/12 (58.3)	3.25 ± 1.12	1	0/12	0	ND
			MFA	27/46 (58.7)		8/12 (66.7)	2.83 ± 0.2		0/12	0	
15	1	5.64	DSFA	16/17 (94.1)	0.66	11/11 (100)	5.21 ± 3.00	0.14	3/12 (27)	4.19 ± 0.57	ND
			MFA	37/42 (88.1)		9/12 (75)	3.87 ± 2.01		1/11 (9)	3.95	
16	1	8.85	DSFA	17/17 (100)	1	12/12 (100)	9.50 ± 0.11	0.02*	12/12 (100)	4.64 ± 0.49	0.8
			MFA	46/46 (100)		12/12 (100)	9.66 ± 0.17		12/12 (100)	4.34 ± 0.67	
17	1	4.75	DSFA	17/17 (100)	0.02*	8/12 (66.7)	2.62 ± 0.01	0.16	0/12	0	ND
			MFA	40/56 (71.4)		7/12 (58.3)	3.48 ± 0.82		0/12	0	
18	1	7.55	DSFA	17/17 (100)	0.03*	12/12 (100)	7.6 ± 3.68	0.06	1/12 (8)	4.95	ND
			MFA	42/56 (75)		12/12 (100)	5.44 ± 3.29		6/12 (50)	3.73 ± 0.30	
19	1	7.31	DSFA	17/17 (100)	0.02*	10/12 (83.3)	5.9 ± 3.26	0.45	5/12 (45)	4.69 ± 0.74	0.76
			MFA	39/54 (72.2)		10/11 (90.9)	6.89 ± 3.17		3/12 (25)	4.52 ± 1.00	
20	1	8.2	DSFA	17/17 (100)	0.32	12/12 (100)	9.66 ± 0.24	0.15	12/12 (100)	4.25 ± 0.49	0.54
			MFA	50/56 (89.3)		12/12 (100)	9.51 ± 0.18		12/12 (100)	4.36 ± 0.52	
21	1	8.79	DSFA	17/17 (100)	0.19	12/12 (100)	8.86 ± 0.72	0.4529	12/12 (100)	4.22 ± 0.41	0.63
			MFA	49/56 (87.5)		12/12 (100)	9.12 ± 0.22		12/12 (100)	4.26 ± 0.59	
22	1	8.84	DSFA	17/17 (100)	0.02*	12/12 (100)	9.17 ± 0.12	0.05	12/12 (100)	5.02 ± 0.34	0.033
			MFA	40/56 (71.4)		12/12 (100)	9.37 ± 0.27		12/12 (100)	4.52 ± 0.65	
23	1	9.39	DSFA	17/17 (100)	0.58	12/12 (100)	9.05 ± 0.21	0.1489	12/12 (100)	3.58 ± 0.71	0.42
			MFA	51/56 (91.1)		12/12 (100)	9.14 ± 0.41		12/12 (100)	3.9 ± 0.44	
24	1	6.31	DSFA	17/17 (100)	0.17	12/12 (100)	5.58 ± 2.83	0.8399	3/12 (17)	3.84 ± 1.33	ND
			MFA	42/56 (75)		12/12 (100)	6.1 ± 2.85		6/12 (50)	4.40 ± 0.39	
25	1	8.12	DSFA	17/17 (100)	1	12/12 (100)	9.26 ± 0.46	0.5637	12/12 (100)	4.75 ± 0.58	0.29
			MFA	55/58 (94.8)		12/12 (100)	9.34 ± 0.26		12/12 (100)	4.48 ± 0.46	
26	1	8.03	DSFA	17/17 (100)	1	12/12 (100)	9.03 ± 0.34	0.3865	12/12 (100)	4.99 ± 0.50	0.4
			MFA	55/56 (98.2)		12/12 (100)	8.16 ± 2.26		10/12 (83)	5.15 ± 0.41	
4	2	5.6	DSFA	29/30 (96.7)	1	7/12 (58.3)	3.46 ± 2.34	1	1/12(8.3)	4.52	ND
			MFA	55/56 (98)		7/12 (58.3)	3.52 ± 2.12		1/12(8.3)	2.52	
7	2	3.73	DSFA	29/29 (100)	0.09	11/12 (91.7)	3.04 ± 0.52	0.21	0/12	0	ND
			MFA	51/58 (87.9)		9/12 (75)	3.42 ± 0.65		0/12	0	
1	3	3.69	DSFA	30/30 (100)	0.15	1/12 (8.3)	2.47	ND	0/12	0	ND
			MFA	47/56 (83.9)		2/12 (16.7)	3.11		0/12	0	
2	3	3.94	DSFA	30/30 (100)	<0.0001*	3/12 (25)	1.68 ± 0.38	ND	0/12	0	ND
			MFA	39/59 (69.6)		2/12 (16.7)	1.41		0/12	0	
3	3	8.69	DSFA	30/30 (100)	0.0001*	12/12 (100)	9.23 ± 0.18	0.15	12/12 (100)	5.22 ± 1.26	0.16
			MFA	40/60 (66.7)		12/12 (100)	9.34 ± 0.18		12/12 (100)	4.54 ± 1.14	

^a^Fisher's Exact Test (*P-* value < 0.05 are highlighted*), ^b^Pairwise comparison of midgut and salivary glands infection rate from each patient showed no significant differences by Fisher's Exact Test, *ND* Not done

### Feeding rates

Overall, DSFA consistently resulted in higher mosquito feeding rates when compared to membrane feeding (Table 1). In 11 out of 25 feeding events, a significantly higher number of mosquitoes was found to be fully engorged when they fed directly on a patient’s arm as compared to those fed on the EDTA blood through a membrane (*P* ≤ 0.05). Direct skin feeding consistently achieved more than 90 % feeding rates, while the rate for membrane feeding were between 48.2 and 98.2 %.

### Human to mosquito transmission

#### Midgut infection

**Fig. 1:**
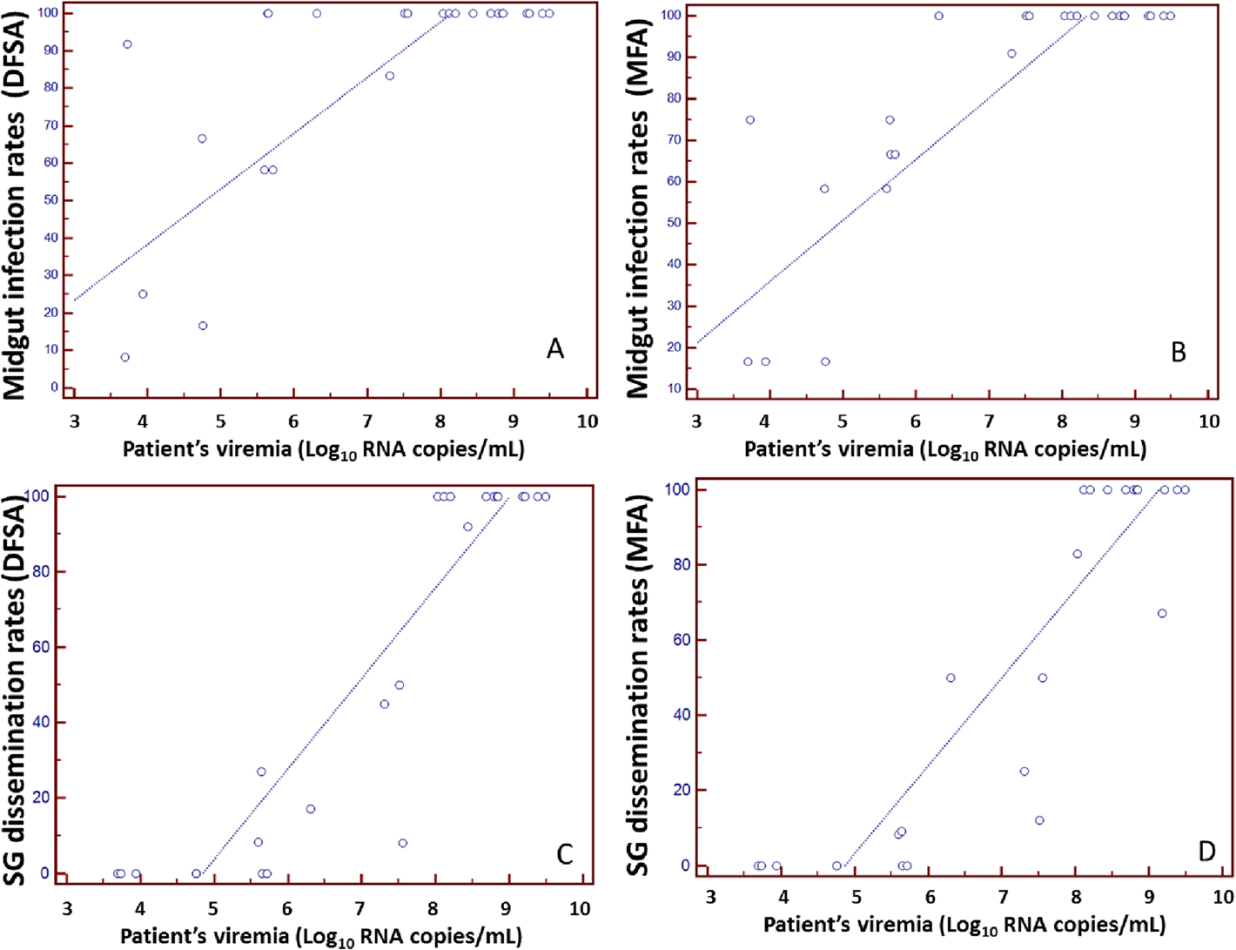
Association between patient serum DENV viremia and percentage of *Ae. aegypti* becoming infected (midgut infection rates) and those that having disseminated infection (salivary gland infection rates) after directly feeding on 25 patients (**a**, **c**) or patient’s EDTA blood fed by membrane feeding assay (MFA) (**b**, **d**). MG – midgut, SG – salivary glands

All feeding experiments, regardless of the feeding method and patient viremia levels, resulted in at least one infected mosquito (Table 1). Pairwise comparisons between DSFA and MFA from each patient revealed no significant differences in midgut infection rates (Table 1). Strong correlation in midgut infection rates was observed between DSFA and MFA (*r* = 0.89, *P* < 0.0001). Strong correlations between midgut infection rates and patient serum viremia was also observed for both DSFA (*r* = 0.72, *P* < 0.0001) and MFA (*r* = 0.84, *P* < 0.0001) (Fig. 1a, b).

**Fig. 2:**
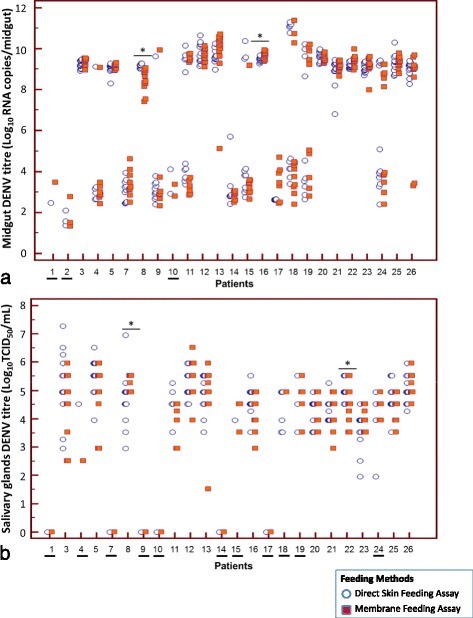
**a** Midgut DENV titre in *Ae. aegypti* fed directly on patients (*blue circles*) and those fed patient’s EDTA blood via membrane feeding assay (MFA) (*red squares*). Midgut DENV titre were expressed as Log10 copies/midgut. *denotes significant difference, *P* < 0.05. Each point represents an individual midgut. Note: Due to small sample size, Mann–Whitney *U*-tests were not conducted for underlined patients’ numbers (1, 2 and 10). **b** DENV salivary gland titre in *Ae. aegypti* fed directly on patients (*blue circles*) and those fed patient’s EDTA blood (*red squares*). DENV titres were expressed as Log_10_ TCID50/ml. *denotes significant difference, *P* < 0.05. Each point represents an individual salivary gland. Note: Due to absence of disseminated infection (patient no 1, 2, 7, 9, 10, 14 and 17) or small sample size (4, 15, 18, 19 and 24), Mann–Whitney *U*-tests were not conducted for these feeding events (underlined patients’ numbers)

The midgut viral genome copies from mossquitoes fed directly on dengue patients and those that were artificially fed with the patient’s EDTA blood are shown in Fig. 2a. Overall, midgut viral titres obtained by both methods were comparable except for mosquitoes fed on patients 8 and 16 (*P* = 0.02). Higher midgut viral genome copies were observed in mosquitoes fed directly on patient 8, while the opposite was observed for mosquitoes fed on patient 16’s EDTA blood. A small number of mosquitoes (up to three) fed on patients 1, 2 and 10, were infected (Fig. 2a). Thus, they were not included in the statistical analysis.

#### Salivary gland infection

Pairwise comparison between DSFA and MFA of each patient revealed no significant differences in salivary gland infection rates (Table 1). Strong correlation in salivary gland infection rates was observed between DSFA and MFA (*r* = 0.81, *P* < 0.0001).

A strong correlation between salivary gland infection and patient serum viremia were observed for both DSFA (*r* = 0.80, *P* < 0.0001) and MFA (*r* = 0.79, *P* < 0.0001) methods (Fig. 1c, d). Unlike midgut infection, salivary gland infection was only observed in mosquitoes fed on patients with viremia of more than 5.5 Log_10_ copies/ml (Table 1).

The viral titres of the salivary gland of mosquitoes fed directly on dengue patients and those that were artificially fed with the patient’s EDTA blood are shown in Fig. 2b. Of the 25 feeding events, seven did not produce disseminated infections (Patients 1, 2, 7, 9, 10, 14 and 17). Another five feeding events (Patients 4, 15, 18, 19, and 24) resulted in only up to three mosquitoes with salivary gland infection, in one or both methods. Thus, only salivary gland viral titres from the remaining 13 feeding events were analysed. Overall, the viral titre of the salivary glands from both methods were comparable, except for mosquitoes fed on Patients 8 and 22 (*P* ≤ 0.03) (Fig. 2b). Higher salivary gland viral titres were observed in mosquitoes fed on patient 8’s blood, while the opposite was observed for mosquitoes fed directly on Patient 22.

## Discussion

Direct skin feeding assay is commonly used to measure human malaria infectivity to anopheline vectors and has been the method of choice in measuring the transmission-blocking activity among candidate vaccines and determining the effectiveness of anti-transmission malaria drugs [23, 24]. Due to ethical issues involved in directly exposing human patients to mosquito bites, several studies were previously undertaken to compare the effectiveness between the DSFA and MFA in measuring the transmission potential of *Plasmodium* from patients to vectors [23–28]. Although DSFA mimicked the natural setting in measuring mosquito infectivity, most of these studies concluded that MFA could be used to replace DSFA in situations where direct exposure of patients to mosquito bites was not possible.

Despite the extensive use of DSFA for malaria studies, there are only a few reports on its use for studying the transmission of dengue from patients to *Aedes* vectors [6–8, 29]. The absence of systemic viremic animal models to study dengue transmission has restricted all experimental oral infection of mosquitoes to those conducted artificially. The common methods of infecting *Aedes* mosquitoes with artificial infectious blood meal to determine the competence of these vectors have some limitations. Serial passages of flaviviruses in cell culture may select for viral mutations that may not be normally found in nature and these changes may alter viral phenotypes, such as attenuation of virulence [15, 30–32]. The viral states in a cell line and the host may also differ. Several studies have shown the adaptation and loss of virulence in DENV and other viruses when serially passaged in cell cultures [30, 33–36]. It has been reported that certain modifications of DENV surface antigens occur as a result of the incorporation of cell membrane components into the virus [36]. In addition, blood from a viremic patient may contain host factors that may influence viral profiles and modulate vector competence, and these components may not be present in an artificial blood meal [2]. During the progression of illness in patients and depending on the infecting DENV serotype and previous exposure of the patient to the virus, anti-dengue antibodies (IgM and IgG) will continue to increase and may influence viral profiles in the patient and affect mosquito susceptibility by neutralizing DENV and preventing virus infection of the midgut [37]. These limitations highlight the need for cautious interpretation of results when performing vector competence studies using artificial blood meals or frozen-thawed samples. The use of freshly- drawn venous blood from symptomatic dengue patients for MFA could potentially avoid the disparities described above. In this study, we did not compare the mosquito infection rates between freshly- drawn venous blood and frozen-thawed blood samples. However, we assume that use of frozen-thawed blood samples from dengue viraemic patients may artificially lower the estimate of vector competence. A study conducted by Richards et al. [14], showed that frozen-thawed DENV has diminished infectivity in *Ae. aegypti* when compared to using fresh virus for the infection of mosquitoes.

The type of anticoagulants used may also affect vector competence. The use of heparinized blood lowers the infectivity of DENV4 over the feeding period, while use of defibrinated blood in vector competence studies may inhibit the attachment of virus onto the mosquito’s midgut cells [38, 39]. Although the effect of EDTA on the kinetics of DENV have not been shown, we have conducted extensive study in our laboratory to compare live animals (guinea pigs) and EDTA blood as a blood-meal source for our different mosquito colonies [40]. We have found no difference in terms of survival rates of females, their fecundity, and hatching rates across eight generations. At present, EDTA is routinely used as anticoagulant for blood feeding of our mosquitoes and for our infection work. In our current study, the overall DENV midgut and salivary gland infection results at 13 days post- infection showed good concordance between the two methods, suggesting that MFA using freshly- drawn blood treated with EDTA closely mimicked direct skin feeding.

This study compared exposure of mosquitoes using fresh blood via MFA with DSFA. The main advantage of DSFA is that it consistently showed higher feeding rates (90 to 100 %) when compared to the MFA (48.2 to 98.2 %). This could reflect the strong preference of *Ae. aegypti* to feed on humans. However, the lower feeding rate obtained through MFA can be compensated by increasing the number of mosquitoes used. MFA offers the advantage of potentially higher rate of patient recruitment for such studies and can be performed on residual blood drawn for diagnostic tests. Therefore, it is less intrusive and could reduce the potential risk of allergic reaction to mosquito bites. The advantages offered by MFA outweigh the inconvenience of having to use more mosquitoes to compensate for the reduced feeding rate in MFA. Table 2 summarizes the advantages and disadvantages of both feeding methods.

**Table 2:** Advantages and disadvantages between Direct Skin Feeding Assay (DSFA) and Membrane Feeding Assay (MFA)

	DSFA	MFA
Advantages	• Closely resembles natural feeding • No delay between obtaining patient blood sample and and mosquito feeding • Prevents needle-stick injury • Higher mosquito feeding rates	• Most diagnostic assay require venous blood • Patient more likely to give consent • Fewer ethical considerations/constraints • Allows sampling from wider age-range group (including children) • Allows using large number of mosquitoes and/or different species of mosquitoes • Allows manipulation of viral titre
Disadvantages	• Patient less likely to provide consent • Ethical approval required (difficult or impossible to obtain in some countries) • Exclusion of children based on ethical considerations • Potential exposure of patients to other arboviruses • Risk of experiments being terminated during feeding due to patient’s discomfort	• Delay between sampling and mosquito feeding • Potential exposure of phlebotomist to blood-borne pathogens (needle-stick injury) • Need for patient to undergo needle penetration (potentially aversive in some cases) • Low mosquito feeding rates

Although we observed positive correlations between host viremia levels and midgut infection/salivary gland infection rates in mosquitoes, the rates of salivary gland infection was consistently lower than the midgut infection rates. The lower number of mosquitoes with infected salivary glands than midguts may be due to the presence of a salivary gland infection barrier preventing the dissemination of the virus to these organs; however, the presence of such barriers for dengue viruses in *Aedes* mosquitoes is still highly debated [41]. An alternative explanation for the observed lower infection rates in salivary glands could be due to the cell-based infectivity assay used in the detection of DENV in the salivary glands instead of the more sensitive qRT-PCR assay that was used to detect midgut infection. Although a more sensitive technique, with a lower threshold of detection, dengue RNA concentrations derived from qRT-PCR assay may not directly translate into infectious virus titre in the hosts [42, 43]. The qRT-PCR assay also measures non-infectious immature and defective virions which are not capable of further infection and replication. However, accurate measurement of infectious virus is critical to understanding dengue transmission; thus, the more laborious cell-based infectivity assay was chosen to measure infectious DENV in the most important organ for transmission, the salivary glands.

The current study was dominated by patients with DENV-1 as recruitment was conducted in 2013, when Singapore was in the midst of a DENV-1 outbreak. Hence, most of the patients enrolled in the study were infected with DENV-1 (80 %), the remainder were infected with DENV-2 (8 %) and DENV-3 (12 %). None of the patients enrolled in the study were found to be infected with DENV-4 which is not common in Singapore [44]. Another drawback of the study is the limited number of patients recruited. Despite our best effort to recruit dengue patients, we managed to recruit 26 individuals willing to provide venous blood and allowed themselves to be bitten by mosquitoes. This is in stark contrast to the study conducted by Nguyen et al. [8] whereby large numbers of patients (> 100) agreed to take part in Vietnam.

## Conclusion

To our knowledge, this may be the first report comparing DSFA and MFA using patient venous EDTA blood to infect *Ae. aegypti* with dengue viruses. Our results showed that freshly- obtained venous blood from symptomatic dengue patients for MFA can be effectively used as a substitute for DSFA, with no compromise in midgut and salivary gland infection rates or titres, especially in circumstances where bioethics approval or patient recruitment are difficult to obtain for vector competence studies.

## Additional file

Additional file 1: Figure S1. Patient’s arm inside a glove-box during feeding experiments. (DOCX 25 kb)
