# Supplementary material for: Membrane feeding of dengue patient’s blood as a substitute for direct skin feeding in studying Aedes-dengue virus interaction
Source: Parasit Vectors. 2016 Apr 15;9:211. doi: 10.1186/s13071-016-1469-6 (PMC4833953; doi:10.1186/s13071-016-1469-6)
Supplement: Additional file 1: Figure S1. — Patient’s arm inside a glove-box during feeding experiments. (DOCX 25 kb) [file 13071_2016_1469_MOESM1_ESM.docx]

**Additional Figures**


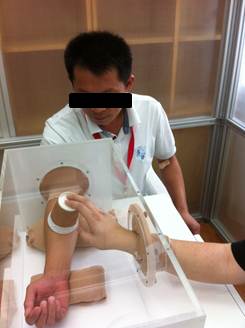


Additional file 1: Figure S1. Patient’s arm inside a glove-box during feeding experiments.
